# Challenges of managing diabetes in Iran: meta-synthesis of qualitative studies

**DOI:** 10.1186/s12913-020-05130-8

**Published:** 2020-06-12

**Authors:** Mohammad Mohseni, Tahereh Shams Ghoreishi, Sousan Houshmandi, Ahmad Moosavi, Saber Azami-Aghdash, Zoleykha Asgarlou

**Affiliations:** 1 Health Management and Economics Research Center, Iran University of Medical Sciences, Tehran, Iran; 2 Department of Midwifery, School of Nursing and Midwifery, Zanjan University of Medical Sciences, Zanjan, Iran; 3 Department of Midwifery, School of Nursing and Midwifery, Ardabil University of Medical Sciences, Ardabil, Iran; 4 Department of Health and Community Medicine, Dezful University of Medical Sciences, Dezful, Iran; 5 Tabriz Health Services Management Research Center, Health Management and Safety Promotion Research Institute, Tabriz University of Medical Sciences, Tabriz, Iran; 6 Khoy University of Medical Sciences, Khoy, Iran

**Keywords:** Systematic review, Qualitative studies, Diabetes, Iran

## Abstract

**Background:**

Although several diabetes management and control programmes are introduced in Iran, many patients do not achieve diabetes-related clinical goals as recommended. The aim of this study was to identify the qualitative evidence for the challenges regarding diabetes management.

**Methods:**

A systematic review of qualitative studies following PRISMA guidelines was undertaken. Scopus, PubMed, Science Direct, and Web of Knowledge were searched as well as Persian databases including Magiran, Irandoc and SID from inception to August 2019. The included Studies were either in English- or Persian-language qualitative studies reporting the perspectives of patients, their relatives, or healthcare service providers. Content of the findings were analysed and organized according to Chronic Care Model framework.

**Results:**

Twelve studies met the inclusion criteria. Six main themes were identified including holistic understanding of patients, leadership and governance difficulties, service delivery, workforce, financing, and information and research.

**Conclusion:**

Challenges regarding the management of diabetes in Iran is multifaceted. Reforming the health care system or developing complementary strategies is essential to improve suitable health care model for patients with chronic conditions such as diabetic patients.

## Background

Diabetes continue to constitute a crucial health issue on a global scale. It is estimated that, only in 2013, approximately 380 million adults were living with diabetes (all types), and this number will rise to 590 million by 2035 [1]. The reports also indicate that regarding the prevalence of diabetes worldwide, the Middle East and North Africa region are ranked 1st [1].

In Iran, as the second-largest country in the Middle East, diabetes is a major public health problem because of its high prevalence rate, increasing incident rate, and economic burden [2]. Mortality associated with diabetes in Iran continue to increase; age standardized mortality rate by diabetes is increased from 8.7 in 2000 to 11.3 in 2015 [3]. Diabetes has also important economic implications in the country. It’s estimated that average per-capita medical cost for Iranian patients with diabetes was equal to $843 only in 2009 [4]. In addition to its high direct health care expenditure, diabetes also is a strong risk factor for other chronic conditions, such as cardiovascular disease [5]. For example, of $843 per capita cost of diabetes, $412 (49%) was related to diabetes complications [4].

It is now well-documented that a good healthcare services and healthy life style can prevent diabetes related complications and enhance the quality of the patient’s life [6]. On the other hand, optimal diabetes management can result in considerable reduction in healthcare expenditure [7]. Although several diabetes management and control programmes are introduced in Iran, the current increasing diabetes related mortality and complication costs reflect suboptimal management of diabetes in the country. According to several reports, Iranian diabetic patients mostly do not get required qualitative services [8] and their usually have poor metabolic (glycemic) control [9, 10]. For example, a nationwide prospective analysis of data for 30,202 patients found that only 13.2%of patients with diabetes achieved satisfactory glycemic control target [11]. This finding reveals challenges regarding current diabetes management in Iran.

Identifying the challenges in diabetes management is important so that policymakers can plan and intervention with it based on the current evidences. In this regards, qualitative approaches can address this subject deeply [12]. Regarding the management of diabetes in Iran, several studies have been conducted to identify major challenges. To the best of our knowledge, there are no qualitative systematic review that address challenges of diabetes management in Iran from perspectives of main stakeholders. Thus, the objective of this study is to acquire a more comprehensive understanding of patients’, their relatives’, or their healthcare providers’ perspective on the challenges that Iranian health system faces regarding managing diabetes. The results of this systematic review will help healthcare policy makers and providers to develop policy and interventions in improving diabetes management that are aligned with key stakeholders’ perspectives.

## Methods

A systematic literature review and evidence synthesis of qualitative evidence on challenges of diabetes management in Iran was conducted based on the Preferred Reporting Items for Systematic Reviews and Meta-Analyses (PRISMA) [13]. Systematic search of Scopus, PubMed, Science Direct, and Web of Knowledge was made, in addition to three Iranian databases (MagIran, Irandoc and Scientific Information Database (SID) databases), from inception to August 2019. The reference lists of the relevant studies were also hand-searched to capture additional potentially relevant citations. The retrieved records were handled using Endnote V.X5. The search strategy combined subject heading/keyword searches for “diabetes”, keyword search for “Iran” and search filters for retrieving qualitative studies recommended by McMaster University Health Information Research Unit [14]. An example search strategy is presented in Appendix.

Peer-reviewed studies that were published in English/Persian and provided original qualitative data regarding the perspectives of patients with diabetes (all types), their relatives, or healthcare providers/policy makers on diabetes management challenges were included. Studies that either concerned with a specific dimension of diabetes management (such as self-care or a diabetes related specific intervention/policy), or more general aspects of diabetes management were included. On the other hand, quantitative studies and papers with closed-ended questionnaires were excluded. One of the authors (MM) conducted the search and eliminated duplicated records. Next, two of the authors (SH and ZA) independently screened the titles of retrieved citations and eliminated irrelevant studies. The reviewers independently evaluated the abstracts of the remaining studies and possible relevant studies remained for further assessment. In the next step, each reviewer read the full-text of the remaining studies for inclusion of citations considered as definitely meeting the eligibility criteria. Disagreements were resolved via a discussion between reviewers.

All included articles were critically appraised by Critical Appraisal Skills Programme (CASP) tool [15]. In this review, two of the researchers (AM, TSG) assessed each study’s quality independently, and after conferral of findings and scores, a decision was made about inclusion.

A pre-piloted data extraction table was used to extract the included studies’ data. The table includes: name of first author, publication year, study setting, study aim, participants, and key findings regarding pertinent themes relating to diabetes management challenges. To analyse and synthesize extracted data, thematic framework analysis based on the Modified WHO key components of health systems was used [16]. The framework structures key concepts in integrated care for patients with multiple chronic conditions can be used by different stakeholders to guide development, implementation, description, and evaluation of health system response regarding chronic conditions. This framework includes: holistic understanding of the patients, service delivery, leadership & governance, workforce, financing, technologies & medical products, and information & research.

Extraction was carried out by (MM). Another reviewer (SAA) looked on the extraction process in more detail to ensure suitability of data extraction. Differences in judgments were resolved by a discussion between reviewers.

## Results

**Fig. 1:**
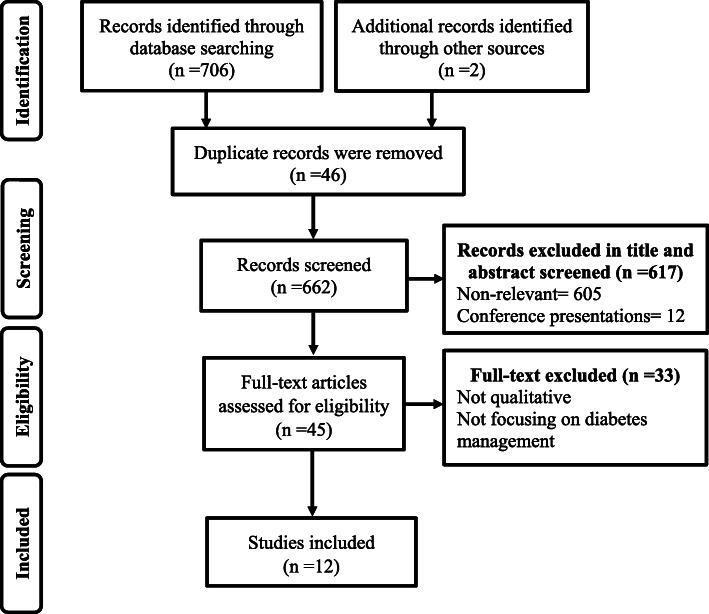
PRISMA flow diagram of identification and selection of literature

The search result yielded a total of 708 studies. After removing 46 duplicates, the title and abstract of the remaining 662 studies were screened for relevancy. Six hundred seventeen obviously irrelevant studies were excluded. Forty five citations were selected for full-text review, of which 12 studies were included. Figure 1 describes the details of literature identification and selection process.

### Overview of the included studies

Included studies were published between 2009 and 2019. Five articles [17–21] focused on patients, two [22, 23] on healthcare providers/experts, and five on mixture of these groups. The total population sample included 188 patients, 2 relatives, and 25 healthcare providers/experts. Nine studies [17–19, 22, 24–27] used face to face qualitative interviews, one [21] used focus group discussions, one used mixture of interviews and focus group discussions [28], and one used mixture of interviews and document review [23].

### Quality of studies

The overall quality assessment of included studies was performed by rating CASP items (Table 1). All of the included studies had a clear statements of the research objectives and appropriate qualitative methodology and descriptions of data analysis techniques were generally provided.

**Table 1:** Overview of included studies

First author /year	Setting	Objectives	Study design	Sampling/ participants	Analysis approach	Key emerged themes	CASP score
Molayaghobi [18], 2019	A specialized polyclinic-Isfahan	To implement, determine, and solve the challenges of executing the Chronic Care Model in diabetes management	Action research/ semi-structured interviews	17 patients with type 2 diabetes.	Content analysis	* lack of effective follow-up system * insufficient acre providers * incomplete medical records * limited knowledge of healthcare providers * lack of regular physician visit * lack of commitment to ordered regime	8
Aliasgharpour [24], 2012	A big university affiliated hospitals in Tehran	to clarify the care process for Iranian patients with diabetes suffering from diabetic foot ulcer condition	Grounded Theory /semi-structured interviews	11 diabetic patients with food ulcer, 4 physicians, one head nurse and one nurse	Theoretical analysis	* disease management * disease experience * continuity of care	9
Shakibazadeh [21], 2011	A diabetes clinic in Tehran	to exploreexperience of Iranian diabetic patients regarding barriers to and facilitatingfactors for diabetes self-care	phenomenological study/ focus groups	43 type 2 diabetic patients	Framework analysis	* physical barriers * psychological barriers * educational barriers * social barriers * care system barriers	8
Ravaghi, 2014 [23]	General (health system)	To evaluate the planning and establishing of the specialized care program for diabetic’s patient from the healthcare providers’ perspectives	documents review and face-to-face semi-structured interviews	Program leaders and relevant executive managers of the local medical universities	Thematic analysis	* program planning * program implementation * Program results	7
Abdoli, 2009 [17]	Hospitals, diabetes clinics, physician offices, and health houses	To identify barriers to and facilitators of empowerment in people with diabetes	grounded theory/ in-depth unstructured interviews	11 diabetic patients	Thematic analysis	* negative view about diabetes * ineffective healthcare systems * poverty and illiteracy	8
Nouhjah, 2014 [19]	A diabetes clinic/ Ahwaz	to explore diabetic women’s views about the diabetes and its consequences	Semi-structured interview	40 Diabetic Women’s	Thematic analysis	* unavailability of drugs * high treatment cost * fear and embarrassment of insulin injection * self-treatment	7
Abazari, 2012 [22]	Ministry of health, and officials of the health system in Isfahan	to describe the situation of training of general practitioners who provide diabetes care	unstructured interviews	8 diabetes management planners and six general practitioners workingin diabetes centers	content analysis	* unstructured education of healthcare provides * inadequate physician’s competence * ineffective education	8
Mousavizadeh, 2018 [26]	Hospitals, diabetes associations, physicians’ offices, patients’	to explore how adherence to diabetes treatment process occurs among Iranian patients	grounded theory/ Semi-structured in-depth interviews	21 diabetic patients+ two members of families and healthcare providers	Corbin and Strauss constant comparative analysis	* unperceived threat in diagnosis time * bitter belief * adaptation to treatment	8
Valizadeh, 2017 [27]	Diabetes centers, treatment and health deputies, and health departments	to identify the problems of Type 2 diabetes prevention and control program in Iran	The semi-structured interview	7 diabetes experts, and 10 diabetic patients	Framework analysis	* Referral system issues * human resources issues * Infrastructure issues * cultural problems issues * access issues * intersectoral coordination issues	9
Dehghani Tafti, 2015 [28]	Health network/ Ardakan	to explore the barriers and incentives for diabetes self-care	focus group discussions and interviews	Diabetic patients and health care providers	content analysis	* product issues * place issues * price issues * promotion issues	8
Molayaghobi, 2019 [25]	A specialized poly-clinic/ Isfahan	To identify diabetes management challenges in Iran	semi-structured interviews	4 members of clinic diabetesunit and 21 type 2 diabetic patients	content analysis	* weak care delivery system * Defective diabetes self-care	9
Rezaei, 2019 [20]	A diabetes unit / Kurdistan	To identify barriers of medication adherence in Iranian patients with type-2 diabetes	Semi-structured interviews	12 patients with type-2 diabetes	conventional content analysis	* disbelief in medical explanatory/prescriptive knowledge * lived experiences of the disease * challenges of everyday life * interactive/economic challenges	9

### Emerged themes

Our findings regarding the challenges of diabetes management in Iran reported below, using the seven dimensions of modified WHO components of health systems (Holistic understanding of the patients, service delivery, leadership and governance, workforce, financing, technologies and medical products) as a guide (Table 2).

**Table 2:** Data extraction by thematic analysis using the modified WHO key components of health systems framework and original themes from included articles

WHO components of health systems	Original sub-themes extracted from studies	Studied themes	
		Major themes	Major themes
Holistic understanding of patients in his/her environment	- Insufficient attention/training from the therapeutic team [24] - Lack of time spent with clients by physicians [21] - Lack of belief among patients in services provided by general practitioners (first level) [23]. - Lack of physicians’ success in earning patients trust [22] - Mistrust of health care providers’ advice [21] - Lack of mutual understanding about patient situation between patients and family members [24] - Being ignored by family members [20] - Lack of support from family [20] - Medically inappropriate expectations of relatives (e.g. To eat more with them) [21] - Patients feel embarrassed injecting insulin in public [21] - Insufficient awareness of public regarding the diabetes [21, 27] - Lack of appropriate programs in media [21] - Lack of free exercise facilities at public parks could be an asset [21] - High living costs resulted in stresses that did not allow diabetes to be as a priority [21]. - Weak organization and performance of NGOs [27]	Holistic understanding of patients in his/her environment	- Insufficient attention to patients - Lack of patients trusts in healthcare and healthcare providers - Insufficient family support - Insufficient community support
Leadership and governance	- Ineffective inter-sectoral coordination (e.g. Health and treatment deputy [23] - Lack of integrated care [23] - Low cooperation of other service providers [27] - Ineffective healthcare systems [17] - Physical separation of first and second level health centers [27] - Incompliance of health network with non-communicable diseases [27] - Weak performance evaluation [27] - Lack of continuous supervision on evidence based instruction performance [25]	Leadership and governance	- Ineffective care coordination - Weak performance evaluation
Service delivery	- Insufficient facilities [24] such as shortage of specialists centers [24] diabetic foot management canters [27] - Lack of support in terms of access to services [21] - Unavailability of drugs [19] - There are no local services [21] - Insufficient laboratory services for thesecond level patients [27] - Unavailability of services [28] - Lack of care at hospital despite timely hospitalization [24] - Long hospital waiting times [24]. - Difficulties in receiving service from public centers [27] and unsuitable working hours [27] - Overcrowded hospitals and outpatient clinics [23] - Problems accessing modern treatments and technologies [21] - Inadequate packages and guidelines [23, 28] - And specialized protocols [23] - No need-based organization chart [25] - Unsuitable health care services for diabetic patients [28] - Patients ignore self-care [26] and have low motivation in this regards [27] - Patients do not consider diabetes as a serious health threat [26] - Patients are not committed to visit the physician regularly [18, 25] and timely [24], to follow ordered nutrition and drug use and regular exercise [18, 24, 25] - Lack of self-efficacy to change lifestyle [21] and difficulties in integrating treatment with daily activities [26] - They avoid to take insulin because they considered insulin consumption as a symptom of their disease deterioration [24] - Most patients did not do regular tests for controlling their blood sugar level [24] - Lower priority of diabetes management compared to other needs (e.g. Children need) [21] - Patient education/training is inadequate [18, 21, 24] - Patients’ poor knowledge and skill regarding the disease [26] - Lack of resources to educate patients [21] - Patients are not committed to participate in group training courses [25] - Misconceptions about diabetes among patients [20] - Patients are unaware of their disease up to appearance of an ulcer [24] - Insufficient information about their nutritious diet [24] and the normal level of blood sugar [24]. - Incomplete information about alternative therapies [21] - Self-medication [19, 20, 24] - Voluntary disorganization of drug use consumption based on self-perception [20] - Negative perceptions of Iranian medicines [21] - Lack of effective follow up system [18, 23–25, 27], especially in elderly patients [27] - Passive referral and lack of coordination in referral [27]. - Low motivation of first level physicians [27]/ High turnover of first level physicians [27]	Service delivery	- Self-management problems: ➢ Lack of patients commitments ➢ Insufficient patients knowledge/training/ skill ➢ Self-medication - Access difficulties - Shortage of diabetes specific facilities - Weak referral system - Inadequate treatment guidelines
Workforce	- Shortage of human resources [24, 27] such as nutritionists [18], nursing [23] - Inadequate knowledge of physicians [22] - Lack of continuous primary training among physicians [22] - Inadequate supervision on physician training process [22] - Lack of integrated education system [27] - Limited interaction among physician and nurses [18] - Lack of shared discussion among the specialists about the curing approach [24] - Lack of physicians’ success in earning colleagues’ trust [22]	Workforce	- Workforce shortage - Insufficient knowledge/training - Weak teamwork
Financing	- Insufficient insurance coverage of first level services [27] - Insurance coverage is not adequate (lack of coverage for blood glucose test strips, glucometers) [21] - Unaffordability of some medicines [27] - Lack of support in terms of cost [21] - High treatment cost [19, 28]	Financing	- Insufficient insurance coverage
Information and research	- Not recording the visit date or the test results [18] - Defective records registration [25] - Information system failure [27] - Inactive information dissemination [23]	Information and research	- Weak information technology
Technologies and medical products	–	–	–

### Holistic understanding of the patients

Overall, participants perceived the lack of a holistic understanding of diabetic patients’ health, abilities, needs and environment. In this regards, four sub-categories emerged: (1) insufficient attention to patients, (2) lack of patients trust in healthcare and healthcare providers, (3) insufficient family support and, (4) insufficient community support.

#### Insufficient attention to patients

Patients highlighted their expectations on the healthcare providers, in relation to feeling listening to and getting attention. They perceived that they receive insufficient attention from the therapy team [24]. Most of participants complained about the insufficient time physician are spending with clients [21].

#### Lack of patients’ trusts in healthcare and healthcare providers

Our results demonstrated the need patients of patients trust in their healthcare professionals. Multiple trust related issues were raised including lack of belief among patients in services provided by general practitioners (first level) [23], lack of physicians’ success in earning patients confidence [22], and mistrust of health care providers’ advice [21].

#### Insufficient family support

The lack of family support was found to be a challenge for patients with diabetes. Several patients reported misunderstanding with family members about their situation [24]. Some of them reported that they are being ignored by family members [20]. Moreover, family members were also seen as a barrier to the diabetes related healthy lifestyles.

#### Insufficient community support

Community support was an area in which frustration was often expressed. The participants believed that there is insufficient awareness of public regarding the diabetes [21, 27]. They criticized the current negative perception of community members’ regarding patients with diabetes, thus they felt embarrassed injecting insulin in public [21]. Moreover, lack of appropriate programs in media was seen as an important sign of poor community support [21]. One of the studies found that lack of free exercise facilities at public parks is a concern for patients with diabetes [21]. High living cost was another sub-domain in this area, preventing diabetes to be a priority [21]. Finally, one of the included studies found poor performance of non-governmental organization (NGOs) another challenge regarding community support [27].

### Leadership and governance difficulties

We found that successful management of diabetes in Iran is hindered by overall health system’s leadership- and governance-related issues. There were two sub-themes related to leadership and governance: Ineffective care coordination and weak performance evaluation.

#### Ineffective care coordination

Participants expected that, as a chronic condition, patients with diabetes should receive the healthcare services through a well-coordinated health system. They were of opinion that provided healthcare service lack integration [23]. They also perceived low cooperation of other service providers [27] and ineffective inter-sectoral coordination (e.g. health and treatment deputy) [23], indicating incompliance of health network with non-communicable diseases [27].

#### Weak performance evaluation

According to the participants’ perspectives, performance assessment in diabetes care is inappropriate. The main challenges are lack of effective [27] and continuous supervision on evidence based instructional performance [25].

### Service delivery

Participants consistently described service delivery constraints as important challenges, especially in relation to self-management practice or general aspects of care. There were five subthemes related to service delivery: (1) self-care management problems, (2) access difficulties, (3) shortage of diabetes specific facilities, (4) weak referral system and, (5) inadequate treatment guidelines.

#### Self-care management problems

According to participants’ point of view, challenges regarding self-care management hinder achievement of good diabetes control. It was determined that “self-management problems” contains three sub-categories: (1) Lack of patients commitment, (2) insufficient patients knowledge/training/ skill, and (3) Self-medication.

The first subcategory of “lack of patient’s commitment” regarding self-management was strongly explained as an important challenge. We found that patients ignore self-care [26] and have low motivation in this regards [27]. Sometime, they do not consider diabetes as a serious health threat [26] or priority [21] and are not committed to visit the physician regularly [18, 25] and timely [24]. Moreover, most of them did not do regular tests for controlling their blood sugar level [24]. In addition, they do not comply with diabetic food/ healthy diet, regular medication regimen and other essential life-style behaviors such as regular exercise [18, 24, 25]. They are also not committed to participate in training/awareness courses related to diabetes [25].

Other self-management barriers includes insufficient patients’ knowledge/training/skill. Patients have no self-efficacy to change their lifestyle (as a critical self-management dimension) [21] and have difficulties in integrating treatment with their daily activities [26]. Most of participants explained that they do not receive appropriate training on self-control [18, 21, 24] that may lead to patients’ poor knowledge and skill regarding the disease and its management [20, 26]. This include insufficient information about appropriate healthy diet [24] normal level of blood sugar [24] and alternative therapies [21]. The issue of poor knowledge of patients was emphasized strongly in some cases; sometimes patients are unaware of their disease processes till their start developing complications [24].

The third area in challenges regarding self- management is concerned with the self-medication [19, 20, 24]. Patients with diabetes, sometimes, do not seek any formal treatments. In this circumstance, they utilize drugs based on their self-perception without any medical command [20]. One of the included studied found that patients’ negative perceptions of Iranian medicines may deteriorate self-medication practice issues [21].

#### Access difficulties

According to participants’ perspectives, access to diabetes care appears inadequate [19, 21, 24, 27, 28]. Sometime, diabetes patients have difficulties in obtaining access to a wide range of services including medications [19], modern treatment and technologies [21], laboratory services [27], service from public centers [27], and local services [21]. They also mentioned that their access to care is restricted by long waiting times in hospitals [24], by unsuitable working hours of outpatient settings [27], or by overcrowded hospitals and outpatient clinics [23]. Patients also explained their experience of lack of access to inpatient care despite timely hospitalization [24].

#### Shortage of diabetes specific facilities

Several participants report inadequate diabetic specific specialist facilities like diabetic foot center as a challenge to those with diabetes [24, 27].

#### Weak referral system

One of the most common mentioned challenge of diabetes management in Iran was weak referral system. In this regards, according to participants, the common problems lack of effective referral and follow up system [18, 23–25, 27], especially in elderly patients [27].

#### Inadequate treatment guidelines

In the care provision of diabetes, there is need for using evidence-based guidelines as highlighted by the participants. Some studies found that patients with diabetes do not receive diabetic care in line with evidence based practice and guidelines [23, 28]. They believed that the provided health cares are not organized based on patients’ need [25] and preferences [28].

### Workforce

Included studies highlighted multiple workforce related issues hindering diabetes management in Iran with three subthemes: (1) workforce shortage, (2) insufficient knowledge/training and, (3) weak teamwork.

#### Workforce shortage

Several respondents expressed concerns over shortage of human resources [24, 27] such as nutritionists [18] and nurses [23].

#### Insufficient knowledge/training

Inadequate knowledge and training of healthcare providers inform of continuous medical education/training was reported as an important barrier to better management of patients with diabetes. Participants perceived lack of continuous [22] and integrated [27] training among physicians, inadequate supervision on physician training process [22], and therefore their inadequate knowledge [22].

#### Week teamwork

Team work issues were reported by several participants as important challenges of managing diabetes in Iran. According to participants’ point of view in including studies, there is not an optimal cross-disciplinary relationship among healthcare providers. They believed that there are limited interactions among physician and other healthcare providers including nurses [18]. In terms of physician-physician relationship, participants mentioned that patients experience a treatment condition that lacks optimal shared discussion among the specialists about the curing approach [24]. Participants cited that physicians fail in earning colleagues’ trust [22].

### Financing

We found that financial support is a key issue that participants care about. In this regards, only one subtheme emerged and is discussed below:

#### Inadequate insurance coverage

Insufficient health insurance, including the insufficient insurance coverage of first level services [27] and lack of coverage for diabetes related equipment and test (e.g. blood glucose test strips, glucometers) [21], associated financial strain of some expensive medications and treatments [19, 21, 28], was reported by participants.

### Information and research

Regarding the information and research dimension, only one subtheme emerged and is discussed below:

#### Weak information technology

Findings of this research indicate that systematic recording of individual level health and healthcare data and utilizing them in care process is one of the important concerns of diabetes care stakeholders. In this area, participants perceived information system failure [27] and poor records registrations [25] that act as an obsolete information technology [23]. They believed that important piece of data such as visit date or the test results are not been recorded [18].

## Discussion

This meta-synthesis sought to provide a comprehensive understanding of diabetes management challenges from the perspectives of the main stakeholders of diabetes care in Iran. The review identified six major themes regarding to diabetes management in the country including: holistic understanding of patients, leadership and governance, service delivery, workforce, financing, and information and research. The results of this study covered broad dimensions of the modified WHO key components of health systems. However, none of the included studies actually addressed the “Technologies and medical products” theme of the modified WHO key components of health system.

In the present review, a wide variety of challenges regarding diabetes management were identified, in which many of them were similar to challenges identified in other studies around the world [29–31]. However, some cultural/context specific barriers in diabetes management were identified such as counter to self-care expectations of relatives (e.g. to eat more with them) [21], lower priority of diabetes management compared to other needs (e.g. children need) [21], and Self-medication [19, 20, 24].

Findings from the included studies determined that many of diabetic patients and their preferences and needs are not being understood appropriately. We found that teamwork is strikingly absent in care provision process of diabetes management. Our review suggests that knowledge barriers were commonly reported by participants. Participants complained about lack of diabetes management skills/knowledge among patients, their relatives, and even healthcare providers. This results, however, is in contrast with Moser et al. [32] that found older adults with type 2 diabetes had a good control over their blood glucose and they followed the recommended nutritional diet; they receive effective training support from diabetes specialists and find answers to their caring questions.

Medication related issues comprised a wide list of emerged sub-themes, of which Insulin utilization was frequently highlighted in included studies. We found that insulin is underutilized among Iranian diabetic patients. This finding supports Sarayani et al. results [33]. In a time-series study of pharmaceuticals wholesale data for Iran, they found that insulin utilization only comprised 17% of total antidiabetic consumption in 2012 [33]. This is very low compared to European countries; share of insulin utilization in European countries was 30% in 2003 [34]. Possible explanations of this underutilization in Iran could be inadequacy of physicians’ knowledge about clinical guidelines as well as patients’ concerns about insulin injection [21, 35].

The national model of non-communicable diseases prevention and control has yet to be implemented [36], and therefore the findings of this review afford opportunity to anticipate the main difficulties and plan solutions taking into account of the identified challenges. First, the results of our review suggest that educational interventions (for patients and their relatives and healthcare providers) may facilitate diabetes care management. Second, partnership that is built up between healthcare providers and between patients and their care givers may facilitate diabetes management. In practice, health care system should aim for continuity of care so the opportunity occurs regarding better information sharing between patients and their physicians [37], enhanced understanding of the patient’s needs [38], and earlier detection and management of serious disease outcomes [37]. In addition, we found that sometimes medication access is limited by insurance companies. Pharmacy benefit management strategies of health insurers need to be changed to facilitate access to diabetes-related medications/equipment.

### Strengths and limitations of the study

This review gathered evidence of diabetes management difficulties in Iran from the perspective of a wide involved groups, namely patients, their relative, and healthcare providers/policymakers, which can help systematic management of diabetes care. In addition, in this review, we focused on qualitative studies to provide an in-depth exploration of the issue. However, this study have some limitations that should be considered when interpreting the results. First, decisions about the search strategy and database selection may influence the retrieving of all of the relevant articles [39]. Another limitation is the fact that this review addresses only those challenges reported by participants that may not be acknowledged of all potential barriers regarding diabetes management in Iran.

## Conclusion

The results of this systematic review are evidence for existence of fundamental weaknesses in diabetes management in Iranian healthcare system. The modified WHO key components of health systems are reflected in the emerged challenges of this study. Iranian healthcare system need a new comprehensive integrated care model for management of chronic health conditions like diabetes, in which the system improves from fragmented, disease-centered, inaccessible care to a patient-centered, holistic and continuous care with healthcare provides alliance. The results of this systematic review can contribute to a better implementation of diabetes management programmes in Iran and similar to many developing countries.

## Data Availability

All data generated or analysed during this study are included in this published article [and its supplementary information files].

## Appendix

### Search strategy

1. diabetes/.exp. or (diabet*, blood glucose or hyperglycemia or glucose or hemoglobin A1c or insulin):ti,ab.
2. interview/.exp. or qualitative research/.exp. or (qualitative study or qualitative research or focus-group* or experience* or attitude): ti, ab.
3. Iran or Iranian or Persia.
4. #1 AND #2 AND #3.

## Abbreviations

PRISMA: Preferred Reporting Items for Systematic Reviews and Meta-Analyses
SID: Scientific Information Database
CASP: Critical Appraisal Skills Programme
WHO: World Health Organization
NGO: Non-governmental Organization

## References

[1] Guariguata L, Whiting DR, Hambleton I, Beagley J, Linnenkamp U, Shaw JE (2014). Global estimates of diabetes prevalence for 2013 and projections for 2035. Diabetes Res Clin Pract.

[2] Esteghamati A, Khalilzadeh O, Anvari M, Meysamie A, Abbasi M, Forouzanfar M, Alaeddini F (2009). The economic costs of diabetes: a population-based study in Tehran, Iran. Diabetologia.

[3] Veisani Y, Khazaei S, Jenabi E, Delpisheh A (2018). Diabetes mortality and morbidity trends and related risk factors in Iranian adults: an appraisal via current data. J Tehran Univ Heart Center.

[4] Javanbakht M, Baradaran HR, Mashayekhi A, Haghdoost AA, Khamseh ME, Kharazmi E, Sadeghi A (2011). Cost-of-illness analysis of type 2 diabetes mellitus in Iran. PLoS One.

[5] Bonakdaran S, Ebrahimzadeh S, Noghabi S (2011). Cardiovascular disease and risk factors in patients with type 2 diabetes mellitus in Mashhad, Islamic Republic of Iran.

[6] Association AD (2015). Standards of medical care in diabetes—2015 abridged for primary care providers. Clin Diab.

[7] Wan Q, Harris M, Jayasinghe U, Flack J, Georgiou A, Penn D, Burns J (2006). Quality of diabetes care and coronary heart disease absolute risk in patients with type 2 diabetes mellitus in Australian general practice. BMJ Qual Saf.

[8] Shaghaghi A, Ahmadi A, Matlabi H (2014). Iranian patients require more pertinent care to prevent type 2 diabetes complications. Adv Prev Med.

[9] Heshmati H, Behnampour N, Khorasani F, Moghadam Z (2014). Prevalence of chronic complications of diabete and its related factors in referred type 2 diabetes patients in Freydonkenar diabetes center. J Neyshabur Univ Med Sci.

[10] Delpisheh A, Azizi H, Dantalab Esmaeili E, Haghiri L, Karimi G, Abbasi F (2016). The quality of care and blood sugar control in type ΙΙ diabetic patients of rural areas under the care by family physicians. Iran J Diab Lipid Disord.

[11] Esteghamati A, Larijani B, Aghajani MH, Ghaemi F, Kermanchi J, Shahrami A, Saadat M, Esfahani EN, Ganji M, Noshad S (2017). Diabetes in Iran: prospective analysis from first Nationwide diabetes report of National Program for prevention and control of diabetes (NPPCD-2016). Sci Rep.

[12] Refaie Shirpak K, Guruge S, Chinichian M (2010). Meta-synthesis of qualitative research in health sciences. Iran J Epidemiol.

[13] Moher D, Liberati A, Tetzlaff J, Altman DG (2009). Preferred reporting items for systematic reviews and meta-analyses: the PRISMA statement. Ann Intern Med.

[14] Search filters for MEDLINE in ovid syntax and the PubMed translation [https://hiru.mcmaster.ca/hiru/HIRU_Hedges_MEDLINE_Strategies.aspx#Qualitativ].

[15] CASP qualitative checklist [https://casp-uk.net/wp-content/uploads/2018/01/CASP-Qualitative-Checklist-2018.pdf].

[16] Leijten FR, Struckmann V, van Ginneken E, Czypionka T, Kraus M, Reiss M, Tsiachristas A, Boland M, de Bont A, Bal R (2018). The SELFIE framework for integrated care for multi-morbidity: development and description. Health Policy.

[17] Abdoli S, Ashktorab T, Ahmadi F, Parvizi S (2009). Barriers to and facilitators of empowerment in people with diabetes. Iran J Endocrinol Metabol.

[18] Molayaghobi NS, Abazari P, Taleghani F, Iraj B, Etesampour A, Zarei A, Hashemi H, Abasi F (2019). Overcoming challenges of implementing chronic care model in diabetes management: an action research approach. Int J Prev Med.

[19] Nouhjah S, Fayazi F (2014). A qualitative study to define diabetic women’s views about health, illness, complications and experienced restrictions, attending Ahvaz diabetes clinic. Iran J Endocrinol Metabol.

[20] Rezaei M, Valiee S, Tahan M, Ebtekar F, Gheshlagh RG (2019). Barriers of medication adherence in patients with type-2 diabetes: a pilot qualitative study. Diabetes Metab Syndr Obes Targets Ther.

[21] Shakibazadeh E, Larijani B, Shojaeezadeh D, Rashidian A, Forouzanfar M, Bartholomew L (2011). Patients’ perspectives on factors that influence diabetes self-care. Iran J Public Health.

[22] Abazari P, Vanaki Z, Mohammadi E, Amini M (2012). Challenges of training diabetes nurse educator in Iran. Iran J Nurs Midwifery Res.

[23] Ravaghi H, Sajadi HS, Ghotbi M, Sarvarizadeh S, Sharbafchizadeh N, Kermanchi J (2014). Evaluation of an urban phase of the specialized care program for diabetes in Iran: providers’ perspectives. Int J Prev Med.

[24] Aliasgharpour M, Nayeri ND (2012). The care process of diabetic foot ulcer patients: a qualitative study in Iran. J Diab Metab Disord.

[25] Molayaghobi NS, Abazari P, Taleghani F, Iraj B (2019). Diabetes management challenges in Iran: a qualitative content analysis. J Nurs Manag.

[26] Mousavizadeh SN, Ashktorab T, Ahmadi F, Zandi M (2018). From negligence to perception of complexities in adherence to treatment process in people with diabetes: a grounded theory study. Iran J Med Sci.

[27] Valizadeh R, Vali L, Bahaadinbeigy K, Amiresmaili M (2017). The challenges of Iran’s type 2 diabetes prevention and control program. Int J Prev Med.

[28] Dehghani Tafti AA, Mazloomymahmoodabad SS, Morowatisharifabad MA, Khalilzadeh SH, Rezaeipandari H (2015). Barriers and incentives of self-care from the view of diabetic patients and their service providers using the social marketing model in Ardakan, Iran. J Qual Res Health Sci.

[29] Mc Hugh S, O'Mullane M, Perry IJ, Bradley C (2013). Barriers to, and facilitators in, introducing integrated diabetes care in Ireland: a qualitative study of views in general practice. BMJ Open.

[30] Sibounheuang P, Sookanakenun P, Kittiboonyakun P. Patients’ and healthcare providers’ perspectives on diabetes management: a systematic review of qualitative studies. Res Soc Adm Pharm. 2019. 10.1016/j.sapharm.2019.09.001. 10.1016/j.sapharm.2019.09.001 31542445

[31] Alberti H, Boudriga N, Nabli M (2007). Primary care management of diabetes in a low/middle income country: a multi-method, qualitative study of barriers and facilitators to care. BMC Fam Pract.

[32] Moser A, van der Bruggen H, Widdershoven G, Spreeuwenberg C (2008). Self-management of type 2 diabetes mellitus: a qualitative investigation from the perspective of participants in a nurse-led, shared-care programme in the Netherlands. BMC Public Health.

[33] Sarayani A, Rashidian A, Gholami K (2014). Low utilisation of diabetes medicines in Iran, despite their affordability (2000–2012): a time-series and benchmarking study. BMJ Open.

[34] Melander A, Folino-Gallo P, Walley T, Schwabe U, Groop P-H, Klaukka T, Vallano A, Laporte J-R, Gallego M, Schiappa M (2006). Utilisation of antihyperglycaemic drugs in ten European countries: different developments and different levels. Diabetologia.

[35] Peimani M, Tabatabaei-Malazy O, Heshmat H, Sanjari M, Pajouhi M (2010). Knowledge, attitude and practice of physicians in the field of diabetes and its complications; A pilot study. J Diab Metab Disord.

[36] Peykari N, Hashemi H, Dinarvand R, Haji-Aghajani M, Malekzadeh R, Sadrolsadat A, Sayyari AA, Asadi-Lari M, Delavari A, Farzadfar F (2017). National action plan for non-communicable diseases prevention and control in Iran; a response to emerging epidemic. J Diab Metab Disord.

[37] Saultz JW, Lochner J (2005). Interpersonal continuity of care and care outcomes: a critical review. Ann Fam Med.

[38] Hänninen J, Takala J, Keinänen-Kiukaanniemi S (2001). Good continuity of care may improve quality of life in type 2 diabetes. Diabetes Res Clin Pract.

[39] McDonald S, Taylor L, Adams C (1999). Searching the right database. A comparison of four databases for psychiatry journals. Health Libr Rev.

